# Identification of a global gene expression signature associated with the genetic risk of catastrophic fracture in iPSC‐derived osteoblasts from Thoroughbred horses

**DOI:** 10.1111/age.13504

**Published:** 2025-01-13

**Authors:** Esther Palomino Lago, Amy K. C. Ross, Alyce McClellan, Deborah J. Guest

**Affiliations:** ^1^ Department of Clinical Sciences and Services, Centre for Vaccinology and Regenerative Medicine The Royal Veterinary College Hatfield Herts UK; ^2^ Animal Health Trust Newmarket UK; ^3^ Department of Veterinary Medicine University of Cambridge Cambridge UK

**Keywords:** bone, fracture, horse, induced pluripotent stem cells, osteoblasts, RNA sequencing, Thoroughbred

## Abstract

Bone fractures are a significant problem in Thoroughbred racehorses. The risk of fracture is influenced by both genetic and environmental factors. To determine the biological processes that are affected in genetically susceptible horses, we utilised polygenic risk scoring to establish induced pluripotent stem cells (iPSCs) from horses at high and low genetic risk. RNA‐sequencing on iPSC‐derived osteoblasts revealed 112 genes that were significantly differentially expressed. Forty‐three of these genes have known roles in bone, 27 are not yet annotated in the equine genome and 42 currently have no described role in bone. However, many of the proteins encoded by the known and unknown genes have reported interactions. Functional enrichment analyses revealed that the differentially expressed genes were overrepresented in processes regulating the extracellular matrix and pathways known to be involved in bone remodelling and bone diseases. Gene set enrichment analysis also detected numerous biological processes and pathways involved in glycolysis with the associated genes having a higher expression in the iPSC‐osteoblasts from horses with low polygenic risk scores for fracture. Therefore, the differentially expressed genes may be relevant for maintaining bone homeostasis and contribute to fracture risk. A deeper understanding of the consequences of mis‐regulation of these genes and the identification of the DNA variants which underpin their differential expression may reveal more about the molecular mechanisms which are involved in equine bone health and fracture risk.

## INTRODUCTION

Bone fractures in horses can occur owing to a single point traumatic event (such as a kick or fall), but in Thoroughbred racehorses, they often occur in response to high‐intensity, repetitive loading that over time leads to structural failure (Stover, 2013). Fractures owing to bone overloading are the most common musculoskeletal injury in training and racing accounting for 41% of all musculoskeletal injuries (Johnston, Riggs, et al., 2020). Non‐fatal stress fractures lead to a significant loss of time in training, earnings and race starts (Johnston, Sidhu, et al., 2020). However, more complex fractures in horses can be difficult to treat because of the need for continuous weight‐bearing in the injured limb during recovery. As such, fracture is the main reason for euthanasia on the racecourse (McKee, 1995), accounting for approximately 75% of all UK racecourse fatalities (Rosanowski et al., 2018) and an average of 60 horses/year suffering a fatal, catastrophic distal limb fracture during racing in the UK (Parkin et al., 2004a). Fractures therefore have a large economical and welfare impact.

Fracture risk is influenced by both environmental risk factors (Anthenill et al., 2007; Georgopoulos & Parkin, 2017; Kristoffersen et al., 2010; Parkin et al., 2004b; Verheyen et al., 2006) and genetic risk factors. The heritability of fracture risk in Thoroughbreds is 0.21–0.37 (Welsh et al., 2014). A genome wide association study identified significant genetic variation associated with catastrophic fracture risk on chromosomes 9, 18, 22 and 31 and four significantly associated SNPs: three on ECA18 (horse chromosome 18) and one on ECA1 (Blott et al., 2014). Additional SNPs within the region on ECA18 were also found to be associated with fracture (Tozaki et al., 2020). However, the molecular processes affected in horses at high genetic risk and the interplay between genetic and environmental risk factors remain poorly understood. Studies on the pathology of fracture indicate stress‐related damage to the bone prior to catastrophic injury (Stover, 2003). This may be related to abnormal bone repair, as horses euthanised following a fracture show changes in other bones including stress fractures and excessive bone remodelling (Anthenill et al., 2010; Parkin et al., 2006; Riggs et al., 1999; Stover et al., 1992). This suggests that the bone cells involved in tissue homeostasis may be affected in horses that suffer from fatal fractures. However, it has proven challenging to understand the genes and biological pathways that may be involved in fracture risk and studies using bone tissues are complicated by the confounding environmental risk factors.

We have recently developed a genome‐wide polygenic risk score (PRS) for catastrophic fracture (Palomino Lago et al., 2024). Polygenic risk scores provide an estimate of the genetic risk of the phenotypic trait at the level of the individual (Choi et al., 2020) and their potential clinical utility is becoming increasingly evident (Lewis & Vassos, 2020). We previously utilised the PRS for fracture to establish an *in vitro* cell model to study bone gene regulation in cells from horses at high and low risk (Palomino Lago et al., 2024), in the absence of any environmental variability. In that study we used a candidate gene approach and identified that *COL3A1* was differentially expressed between high‐ and low‐risk horses, in part owing to the presence of an upstream SNP that we subsequently demonstrated was significantly associated with fracture using genotying of a cohort (Blott et al., 2014) of 91 phenotyped fracture cases and 86 fracture controls (Palomino Lago et al., 2024).

However, the candidate gene approach only provided insights into a very narrow range of genes and the use of primary cells in this model was associated with technical limitations such as restricted cell growth. Induced pluripotent stem cells (iPSCs) may provide better cell models owing to their ability to differentiate into a wide range of cell types and unlimited growth *in vitro*. They have been used to study many inherited conditions in humans (Fear et al., 2023; Gorashi et al., 2023; Mae et al., 2023) and are of particular value when access to the affected cell types is limited and/or the cells do not expand well in culture (Pereira et al., 2023). More recently human iPSCs have been used in conjunction with polygenic risk scores (Coleman, 2022; Dobrindt et al., 2021; Yde Ohki et al., 2023) to study the mechanisms underlying complex genetic conditions. We have previously derived iPSCs from horses (Bavin et al., 2015; Palomino Lago et al., 2023) and established the methods to differentiate them into bone forming osteoblasts (Baird et al., 2018, 2019). Furthermore, we demonstrated that the genetic background of the iPSCs affects their expression of osteoblast‐associated genes, whereas different iPSC‐lines derived from the same horse had less variance (Baird et al., 2018).

The aim of this study was to use iPSC‐osteoblasts derived from Thoroughbred horses with high and low PRS for catastrophic fracture and perform global gene expression analysis to identify the biological processes and pathways that are perturbed in osteoblasts from high‐risk horses.

## MATERIAL AND METHODS

### Experimental design

**FIGURE 1:**
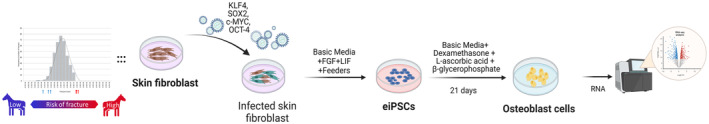
Overview of the experimental approach. Created with Biorender.com. Skin fibroblasts from six Thoroughbred horses at low (blue arrows) or high (red arrows) genetic risk of fracture compared with the general population (382 Thoroughbreds of unknown fracture status) were selected as previously described (Palomino Lago et al., 2024). They were retrovirally transduced to overexpress pluripotency genes *KLF4*, *SOX2*, *c‐MYC* and *OCT4* and clonal lines of induced pluripotent stem cells (iPSCs) were produced and maintained by culturing in the presence of feeders, FGF (fibroblast growth factor) and LIF (Leukaemia Inhibitory Factor). Equine iPSCs (eiPSCs) were induced to differentiate into osteoblasts by 21 days of culture in osteogenic media prior to RNA isolation and sequencing.

An overview of the experimental design used in this study is shown in Figure 1. Frozen stocks of skin fibroblasts that had been derived and banked from six male Thoroughbred horses were used. The skin biopsies were taken at post‐mortem from horses which had been euthanised for reasons unrelated to this study and with the consent of the Animal Health Trust Ethical Review Committee (AHT_02_2012) and Royal Veterinary College Clinical Research Ethical Review Board (URN 2021 2035‐2). The samples had previously been genotyped and polygenic risk scores for catastrophic fracture had been calculated to select samples representing the high and low ends of the risk spectrum. Three samples were from horses at high genetic risk (HR) and three samples were from horses at low genetic risk (LR) (Palomino Lago et al., 2024).

### iPSC generation

Induced pluripotent stem cell lines were generated previously from skin fibroblasts by retroviral transduction using methods previously described (Baird et al., 2018; Bavin et al., 2015). Briefly, phoenix gag‐pol packaging cells were transfected with 3 μg of pVPack‐VSV‐G (Agilent Technologies, UK) along with 3 μg of pMXs.hOct4 (Octamer‐Binding Protein 4, RRID:Addgene_17217), pMXs.hSox2 (SRY‐Box Transcription Factor 2, RRID:Addgene_17218), pMXs.hKlf4 (Kruppel Like Factor 4, RRID:Addgene_17219), pMXs.hc‐Myc (MYC Proto‐Oncogene, RRID:Addgene_17220) or pMX.GFP (Cell Biolabs, USA). Transfections were carried out using lipofectamine3000 and Opti‐MEM media (both Invitrogen, Thermo Fisher, UK) according to the manufacturer's instructions. After 48 h culture supernatant containing the viral particles was pooled, filtered through a 0.45 μm filter (Nalgene, Thermo Fisher), supplemented with 10 μg/mL polybrene (Sigma‐Aldrich, UK) and used to infect equine skin fibroblasts which had been plated at a density of 1 × 10^4^ the day before infection. After three rounds of viral infection, performed at 48 h intervals, infected cells were plated at a density of 5 × 10^3^ cells per 10 cm plate pre‐seeded with feeder cells (mitotically inactivated mouse embryonic fibroblasts). The media was replaced with basic media (DMEM/F12, 15% fetal bovine serum, 2 mm l‐glutamine, 0.1 mm non‐essential amino acids, 0.1 mm 2‐*β*‐mercaptoethanol, 1 mm sodium pyruvate (all from Thermo Fisher)) plus 1000 U/mL Leukaemia inhibitor factor (Preprotech, UK) and 10 ng/mL basic fibroblast growth factor (Peprotech). The iPSC media was replaced every other day until iPSC colonies began to appear and reached a large enough size to manually pick selected colonies. These colonies were used to establish clonal iPSC lines. For each skin fibroblast sample, two or three clonal lines of iPSCs were generated to provide a total of 14 iPSC lines. The iPSCs were mechanically passaged in the presence of 2 μm Thiazovivin (Miltenyi Biotec, UK). All of the equine iPSC clones were previously characterised for their ability to form embryoid bodies, express pluripotency markers and differentiate into derivatives of endoderm, ectoderm and mesoderm (Baird et al., 2018; Bavin et al., 2015). Furthermore, all of the iPSC lines used in this study were differentiated into osteoblasts capable of producing a mineralised matrix and expressing osteoblast associated genes (Baird et al., 2018, 2019; Palomino Lago et al., 2023).

### iPSC differentiation into osteoblasts

Osteoblasts were generated as previously described (Baird et al., 2018). Small pieces of iPSC colonies were plated at a density of 7 × 10^4^ cells per well of a 24‐well OsteoAssay surface coated plate (Corning, Wiesbaden, Germany) without feeders and in iPSC media in the presence of 2 μm Thiazovivin for the first 24 h. The following day, the media was replaced with osteogenic media (DMEM/F12, supplemented with 15% fetal bovine serum, 2 mm l‐glutamine, 1% non‐essential amino acids, 1 mm sodium pyruvate, 0.1 mm 2‐mercaptoethanol (all Invitrogen, Thermo Fisher), 10 mm β‐glycerophosphate, 10 nm dexamethasone and 28 μm ascorbic acid (all Sigma‐Aldrich)). Cells were cultured for 21 days with the media replaced every 2–3 days prior to RNA extraction.

### RNA extraction

Following cell differentiation, RNA was collected using Tri‐reagent (Sigma‐Aldrich) and extracted using the RNeasy Mini Kit (Qiagen, UK) following the manufacturer's instructions. Purified RNA was treated with the DNA‐free™ DNA removal kit (Invitrogen, Thermo Fisher) to remove genomic DNA contamination according to the manufacturer's instructions. RNA concentration was determined using a Qubit (Thermo Fisher Scientific) and the purity was determined using a DS‐11 spectrophotometer to ensure 260/280 ratios ~2.0. RNA integrity was measured using a Tapestation (2100 Bioanalyzer; Agilent, UK) and for all samples was confirmed to be >9.0.

### RNA sequencing

RNA from 14 lines of iPSC‐osteoblasts (passages 8–15) was used in RNA sequencing. mRNA library preparation and transcriptome sequencing were conducted by Novogene (Cambridge, UK) using an Illumina NovaSeq 6000 to generate 26.1–57.2 million 150 bp paired‐end reads per sample. fastqc (v.0.11.9) (Babraham Bioinformatics, Cambridge, UK) and multiqc (v1.11) were used to establish raw sequencing quality. Raw RNA‐sequencing (RNA‐seq) reads were aligned to equine transcriptome obtained from the National Centre for Biotechnology Information (Sayers et al., 2022) EquCab 3.0 annotation release 103 (GCF_002863925.1_EquCab3.0, https://www.ncbi.nlm.nih.gov/datasets/genome/GCF_002863925.1/, BioSample ID SAMN02953672) using the pseudoaligner salmon (v.1.5.2) in Quasi‐mapping‐based mode with GC‐bias correction (Patro et al., 2017). Then, transcript level mappings were imported into rstudio (v.4.2.1) using tximport (v.1.20) (Soneson et al., 2015). Finally, read counts were normalised using deseq2's median of ratios method (Anders & Huber, 2010) and analysed for differential expression using deseq2 v.1.22.2 (Love et al., 2014). Genes with a log_2_ fold change (Log_2_FC) of ±1 and an adjusted *p*‐value (*p*‐adj) of ≤0.05 were considered as differentially expressed. *p*‐Values were adjusted using the Benjamini and Hochberg method in deseq2.

### Functional annotation analysis

To investigate the biological function related to the differentially expressed genes (DEGs), the PANTHER classification system (https://www.pantherdb.org/) v.17.0 was used to functionally annotate genes based on Gene Ontology (GO) terms (BP, biological process; MF, molecular function; CC, cellular component) and pathways. A false discovery rate (FDR) <0.05 was considered to be a statistically significant enrichment. gene analytics from the LifeMap's GeneCards Suite (https://geneanalytics.genecards.org/) was used to perform pathway analysis, with an entity score of >6 being equivalent to a corrected *p*‐value of ≤0.05 and therefore defined as significantly enriched. Pathway analysis was also performed using Qiagen's ingenuity pathway analysis. The results of the GO and pathway analyses were visualised using r (v.4.2.2).

### Protein–protein interaction network construction

Network analysis was conducted using the string (v.10.5) protein network analyser plug‐in of cytoscape (v3.9.1) (Shannon et al., 2003).

### Gene set enrichment analysis

Functional class sorting of all expressed genes, regardless of whether they were significantly differentially expressed, was performed using gene set enrichment analysis (gsea) software (v.4.3.2) based on H (Hallmark gene sets), C2 (curated gene sets): REACTOME, C2:WP, C2:KEGG, C5 (ontology gene sets) gene set collections (MSigDB v.2023.1) (Subramanian et al., 2005). gsea first ranked all expressed genes according to the significance of differential gene expression between the HR and LR of fracture groups. The enrichment score for each gene set is then calculated using the entire ranked list, which reflects how the genes for each set are distributed in the ranked list. Normalised enriched scores (NES) were determined for each gene set. The significant enrichment of gene set cut‐off was |NES| > 1, nominal *p*‐value ≤ 0.01 and FDR < 0.25.

### Weighted gene co‐expression network analysis

Weighted gene co‐expression network analysis (WGCNA; Langfelder & Horvath, 2008) was performed on all expressed genes. The deseq2 (Love et al., 2014) package (v.1.44) was used to perform gene expression level normalisation with the ‘varianceStablizing Transformation’ function. WGCNA package tool (v.1.75) within r software (v.4.4.1) was used to construct the gene co‐expression networks (Langfelder & Horvath, 2008). An optimal soft threshold (*β* = 14) was selected based on the criterion of approximate scale‐free topology. Module detection was performed using the blockwiseModules function. A heatmap was produced to demonstrate the modules most closely associated with fracture risk. Gene significance was used to measure the correlation between gene expression and fracture risk and module membership was used to measure the correlation between each module eigengene and gene expression.

### cDNA synthesis and quantitative RT‐PCR

To validate the RNAseq, quantitative PCR (qPCR) was used to measure a number of genes using the same samples as had been used in the RNAseq. cDNA was synthesised from 1 μg of RNA using the sensiFAST cDNA synthesis kit (Bioline, UK). Two microlitres of cDNA (corresponding to 20 ng) was used in qPCR. Equine specific primers were designed using primerblast (https://www.ncbi.nlm.nih.gov/tools/primer‐blast/index.cgi) and mfold (http://www.unafold.org/) to produce amplicons of 50–150 bp. Primer sequences can be found in Table S1. Quantitative PCR was performed in duplicate using SYBR Green containing supermix (Bioline) on a Bio‐Rad C1000 Touch Thermal Cycler (Bio‐Rad, UK). The PCR cycle parameters were as follows: 95°C (10 min), followed by 45 cycles of 95°C (15 s), 60°C (15 s) and 72°C (15 s). Following this, a melt curve was produced with readings taken every 1°C from 65 to 95°C. Relative gene expression levels were normalised with the housekeeping gene 18S rRNA using the $2^{-\Delta \Delta C_{t}}$ method (Livak & Schmittgen, 2001). Normality of the data was confirmed using a Shaprio–Wilk test and equal variance confirmed using Levene's test of homogeneity. An independent *t*‐test was then used to compare the mean expression of each gene between the LR and HR samples and *p* < 0.05 was considered statistically significant. All analysis was performed using spss (v.28.0; IBM, UK).

## RESULTS

### RNA sequencing reveals there are differentially expressed genes between iPSC‐osteoblasts derived from horses with low and high genetic risk for catastrophic fracture

RNA sequencing was performed on 14 lines of iPSC‐osteoblasts derived from six different horses (three with a low PRS (L1–L3) and three with a high PRS (H1–H3) for fracture) as detailed in Figure 1. Further information on the cells used in this study can be found in Table S2.

**FIGURE 2:**
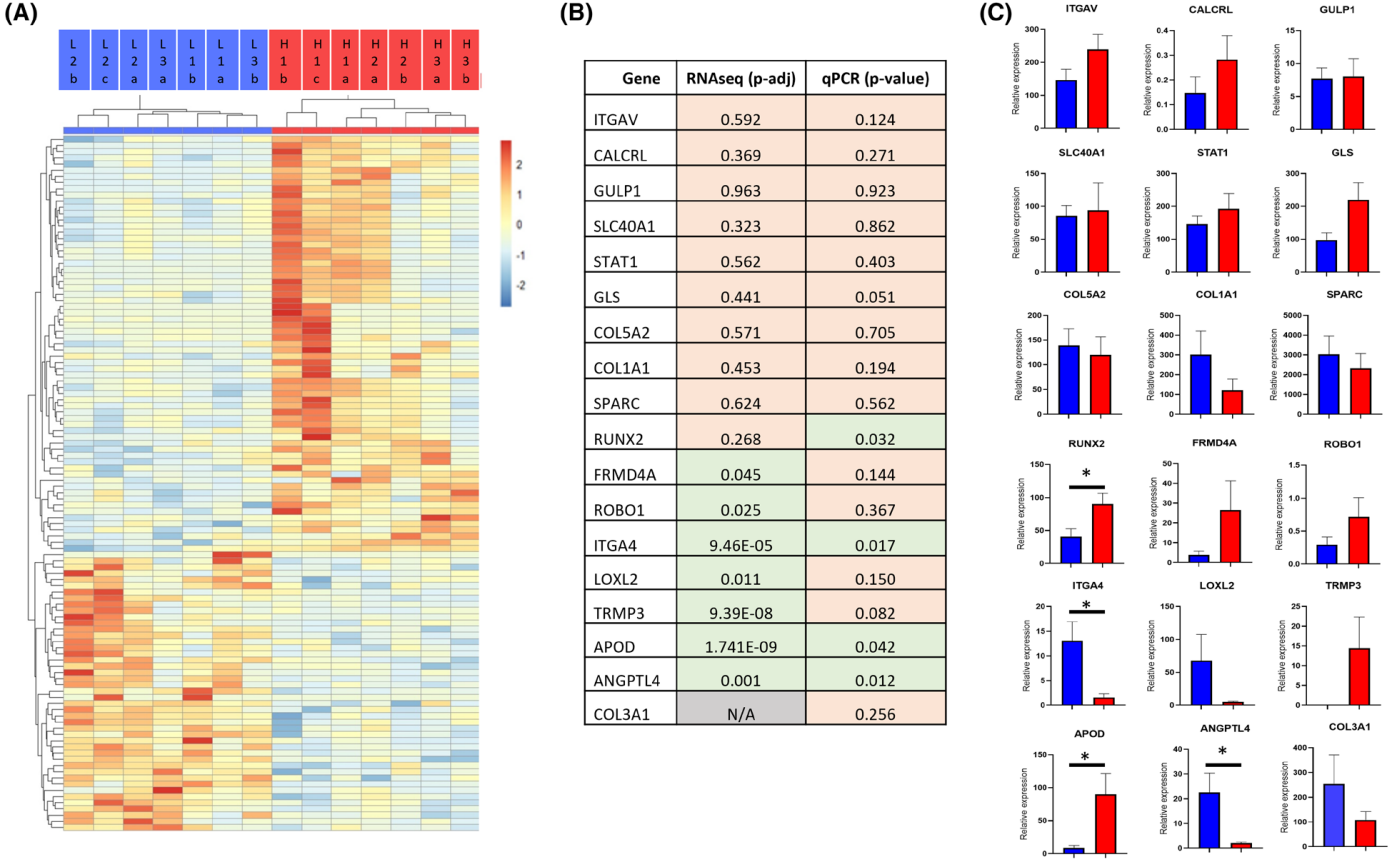
Differential gene expression in induced pluripotent stem cell (iPSC)‐osteoblasts derived from horses at high (HR) and low risk (LR) of fracture. (A) Heatmap showing the 112 differentially expressed genes (*p*‐adj < 0.05 and a log_2_ fold change (FC) of ±1). The samples are shown in columns, three LR horses were used (L1–L3) and three HR horses (H1–H3) (blue = LR samples, red = HR samples). Two or three clonal lines of iPSCs were generated from each horse (a, b, c). Genes are clustered by row (see also Appendix S2). (B) Comparison of differentially expressed genes using RNA‐sequencing (RNA‐seq) and quantitative PCR (qPCR). Genes which are expressed at significantly different levels between HR and LR samples are highlighted in green (*p*‐value; *t*‐test from qPCR data, or *p*‐adj; adjusted *p*‐value from RNAseq data <0.05); those that were not significantly different are highlighted in orange. N/A, Gene not annotated in the reference transcriptome. (C) qPCR data showing expression relative to the housekeeping gene. Blue bars, LR samples; red bars, HR samples. Error bars represent the SEM of seven replicates. *p < 0.05.

RNA sequencing revealed that, of the 29 197 mapped genes, there were 112 DEGs between the iPSC‐derived osteoblasts from horses with a high PRS compared with those with a low PRS (Figure 2A and Appendix S2). To validate the RNA sequencing results, qPCR was carried out to measure the expression of 17 genes (Figure 2B,C). These genes comprised four osteoblast markers (*ITGAV*, *COL1A1*, *SPARC*, *RUNX2*), six genes which showed no significant differences in expression in the RNA sequencing data (*CALCRL*, *GULP1*, *SLC40A1*, *STAT1*, *GLS* and *COL5A2*) and seven genes which did show differential expression in the RNA sequencing data (*FRMD4A*, *ROBO1*, *ITGA4*, *LOXL2*, *TRMP3*, *APOD* and *ANGPTL4*). We also measured the expression of *COL3A1*, which was not annotated in the reference transcriptome but was differentially expressed in our previous study (Palomino Lago et al., 2024). *FRMD4A*, *ROBO1*, *LOXL2* and *TRMP3* showed a significant difference in expression between HR and LR samples in the RNA sequencing data, but no significant difference in the qPCR. However, all genes showed the same trend for differential expression between the samples (Figure 2C). *RUNX2* was expressed at significantly higher levels in the HR horses in the qPCR (Figure 2C) and although it showed the same trend in the RNA sequencing data (average of 2095 normalised counts in HR samples, vs. an average of 859 counts in the LR samples, Appendix S2), this was not significant. Overall, we demonstrated a 71% concordance between the RNA sequencing and qPCR, which is in accordance with other studies (Paterson et al., 2020). Two clusters of differentially expressed genes were observed: those that were significantly more highly expressed in the osteoblasts from HR horses (67 genes) and those that were expressed at significantly lower levels in the osteoblasts from HR horses (45 genes). With the exception of samples from the HR horse 3 (L3a and L3b), all of the samples derived from the same horse clustered together (Figure 2A).

**FIGURE 3:**
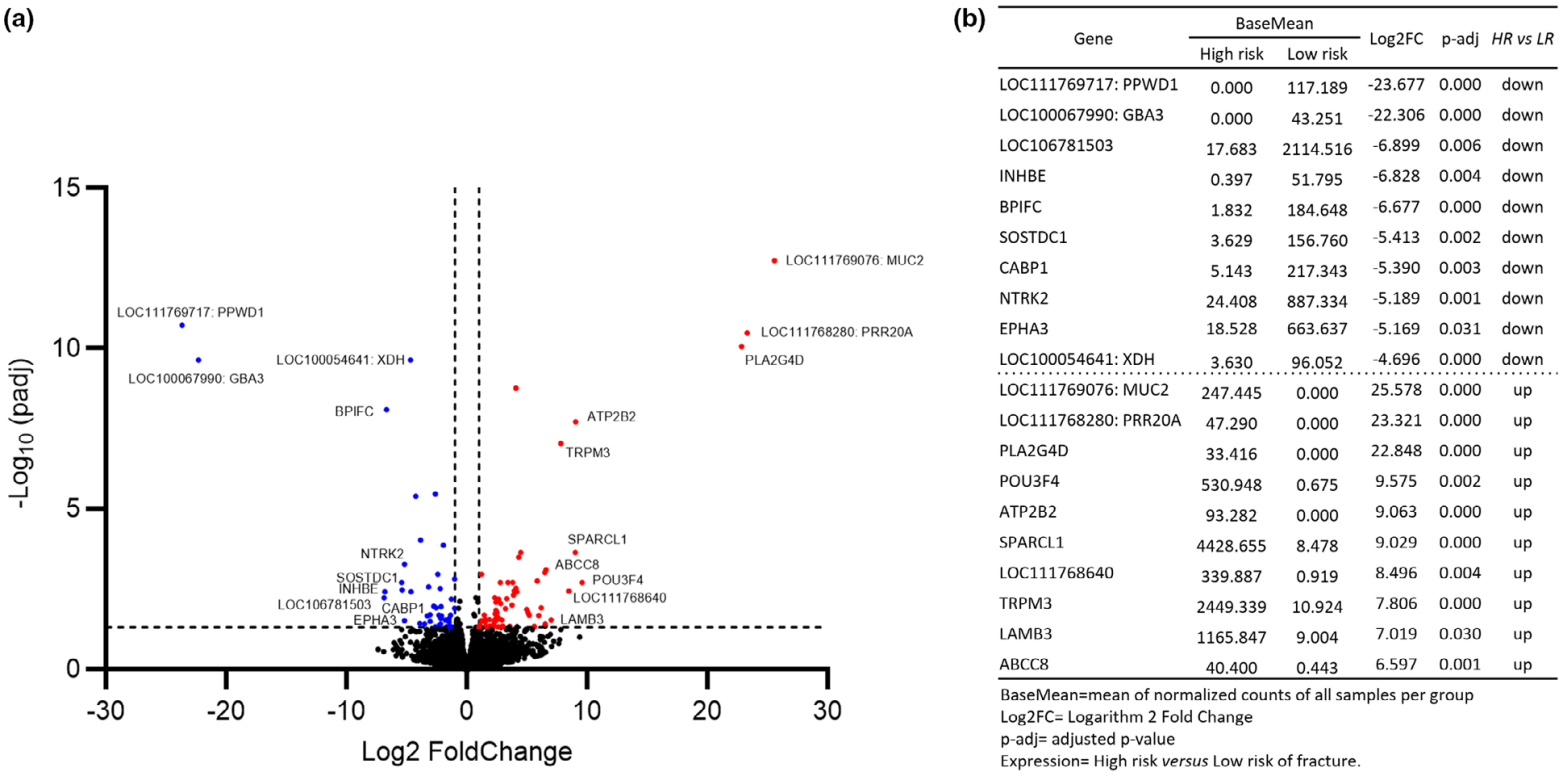
Expression of 29 197 genes in induced pluripotent stem cell (iPSC)‐osteoblasts from high‐risk (HR) compared with low‐risk (LR) horses. (a) Volcano plot illustrating the size (log_2_ fold change, log_2_FC) and significance (*p*‐adj) of the differentially expressed genes. Black dots represent genes that are not significantly different between the groups. Red dots represent genes that are significantly upregulated in the HR samples. Blue dots represent genes that are significantly down‐regulated in the HR samples (*p*‐adj ≤ 0.05 and log_2_FC of ±1). (b) The top 10 differently expressed genes (DEGs) in the HR samples compared with the LR samples. For unannotated genes the predicted orthologues are provided after the identifier where possible (predictions from the National Centre for Biotechnology Information; Sayers et al., 2022).

Of the genes that were downregulated in the HR samples, LOC111769717 (predicted to be *PPWD1*) and LOC1000067990 (predicted to be *GBA3*) had the greatest fold changes (log_2_FC −23.677 and −22.306; HR vs. LR). Of the genes that were upregulated in the HR samples, LOC111769076 (predicted to be *MUC2*), LOC111768280 (predicted to be *PRR20A*) and *PLA2G4D* had the greatest fold changes (log_2_FC 25.578, 23.321 and 22.848, respectively; HR vs. LR) (Figure 3).

### A proportion of the differentially expressed genes have known roles in bone

Of the 112 DEGs, 27 are currently unannotated, 43 have published roles in bone or fracture and 42 have no known role in bone formation of fracture (Table 1). For those genes with a published role in bone or fracture further information can be found in Table 2. The DEGs were distributed across the majority of the chromosomes (Figure S1).

**TABLE 1:** The differentially expressed genes grouped by their known roles in bone, bone formation and/or fracture. C16H3orf58 = DIPK2A (National Centre for Biotechnology Information, NCBI; Sayers et al., 2022). For unannotated genes the predicted orthologues are provided after the identifier where possible (predictions from NCBI; Sayers et al., 2022).

27 Unannotated genes		43 Annotated genes			42 Annotated genes		
		Published role in bone			No published role in bone		
LOC100054641:*XHD* LOC100067768:*UGT3A2* LOC100067990:*GBA3* LOC100069083:*ZNF658* LOC100629234:*GTF2IRD1* LOC102149222 LOC102149317:*RALGDS* LOC102149614 LOC102149688 LOC102150273 LOC102150465 LOC106781042 LOC106781149 LOC106781220:*ABCD2*	LOC106781503 LOC106781645:*PCDHB4* LOC106782354 LOC111767614:*HS3ST4* LOC111768280:*PRR20A* LOC111768640 LOC111769076:*MUC2* LOC111769201 LOC111769717:*PPWD1* LOC111771940 LOC111772113 LOC111774027 LOC111775969	*ABCC5* *ANGPTL4* *APOD* *ATP2B2* *CHAC1* *CHADL* *CNTNAP4* *COBL* *COL11A2* *COL2A1* *EFNB1* *EFNB2* *EPHA3* *EYA4* *FBXO2*	*GPM6B* *HHIP* *HOXD10* *IL15* *ITGA4* *LAMB3* *LAMC2* *LGALS1* *LOXL2* *LRRN3* *MME* *NR2F1* *NR2F2* *NTRK2*	*OGN* *PARVB* *POU3F4* *P2RY6* *RBPMS2* *ROBO1* *ROBO2* *SENP6* *SLC2A3* *SLC38A3* *SMPD3* *SOSTDC1* *SOX10* *TRPM3*	*ABCC8* *ABCG4* *ABI3* *ADGRB1* *ADSSL1* *AMPD3* *ASNS* *BPIFC* *BSN* *C16H3orf58* *CABP1* *CERKL* *CLHC1* *COTL1*	*ENO2* *FRMD4A* *GPHA2* *INHBE* *KIZ* *MATN4* *MUC4* *NAP1L5* *NKAIN1* *NXPH4* *PCDH9* *PEG10* *PLA2G4D* *PLCH2*	*PLPPR3* *PNLIPRP3* *PPP1R2* *PTK7* *SCNN1A* *SCRN2* *SNAP91* *SPARCL1* *SPRED3* *TDRKH* *TMEM38A* *UCP2* *WNK4* *ZSWIM5*

**TABLE 2:** Summary of the differentially expressed genes that have a published role in bone formation or fracture.

Gene	BaseMean	Log_2_ fold change HR vs. LR	*p*‐adj	Chromosome	Highest expression	Role	Reference
*ABCC5*	2427.12	1.04	4.60 × 10^−2^	19	HR	Involved in osteoclast formation	Kang et al. (2020), Mourskaia et al. (2012)
*ANGPTL4*	1453.10	−2.40	1.00 × 10^−3^	7	LR	Stimulates bone resorption, osteoblast proliferation and differentiation	Knowles et al. (2010), Wilson et al. (2015)
*APOD*	1951.83	4.07	1.74 × 10^−9^	19	HR	Deficiency associated with high bone turnover, low bone mass and impaired osteoblast function	Martineau et al. (2016)
*ATP2B2*	46.64	9.06	1.99 × 10^−8^	16	HR	Upregulated during bone loading and part of a family of proteins involved in bone homeostasis	Francis et al. (2002), Kim et al. (2012), Mantila Roosa et al. (2011)
*CHAC1*	171.13	−3.20	2.76 × 10^−3^	1	LR	CHAC1^−/−^ mice have decreased osteoblast differentiation and decreased bone density	Crawford et al. (2016)
*CHADL*	10.31	6.47	1.00 × 10^−3^	28	HR	Increased expression in OA bone; is an OA susceptibility gene	Tuerlings et al. (2021)
*CNTNAP4*	9.25	5.17	2.10 × 10^−2^	3	HR	Involved in Nell‐1 responsive osteogenesis	Li, Zheng, et al. (2018)
*COBL*	1404.20	2.64	7.00 × 10^−3^	4	HR	Contains a fracture‐associated locus	Trajanoska et al. (2018)
*COL11A2*	434.84	5.84	2.00 × 10^−3^	20	HR	Expressed in osteoblasts during bone formation	Urabe et al. (2001)
*COL2A1*	820.86	6.53	4.50 × 10^−2^	6	HR	Mutations associated with skeletal dysplasia with metaphyseal involvement. Bone impairment occurs prior to OA	Rolvien et al. (2020), Walter et al. (2007)
*EFNB1*	3228.79	−1.02	1.30 × 10^−2^	X	LR	Required for bone formation	Cheng et al. (2013), Nguyen et al. (2016), Xing et al. (2010)
*EFNB2*	143.84	2.37	2.90 × 10^−2^	17	HR	Required for normal bone formation	Arthur and Gronthos (2021)
*EPHA3*	341.08	−5.17	3.10 × 10^−2^	26	LR	Part of a family of proteins involved in bone development and homeostasis	Arthur and Gronthos (2021)
*EYA4*	105.62	4.20	5.00 × 10^−2^	10	HR	Gene variants associated with upper limb and skull bone mineral density	Kemp et al. (2014)
*FBXO2*	323.25	3.88	5.00 × 10^−3^	2	HR	Upregulates osteosarcoma cell proliferation via STAT3	Zhao et al. (2020)
*GPM6B*	1543.60	3.95	4.00 × 10^−3^	X	HR	Silencing GPM6B leads to decreased bone differentiation of mesenchymal stromal cells (MSCs)	Drabek et al. (2011)
*HHIP*	300.51	−3.04	3.30 × 10^−2^	2	LR	Increased HHIP inhibits osteogenic differentiation of MSCs	Yin et al. (2022)
*HOXD10*	173.82	2.48	2.30 × 10^−2^	18	HR	Targeted disruption of HOXD10 leads to vertebral and hindlimb bone abnormalities	Carpenter et al. (1997)
*IL15*	705.42	−1.77	2.90 × 10^−2^	2	LR	Pro osteoclastogenic cytokine	Ogata et al. (1999)
*ITGA4*	210.42	−3.86	9.47 × 10^−5^	18	LR	Involved in osteoblast differentiation and mineralisation and regulation of osteoclast activity	Mao et al. (2023)
*LAMB3*	587.43	7.02	3.00 × 10^−2^	5	HR	Part of Laminino‐332 which is produced by osteoblasts and inhibits osteoclast differentiation	Uehara et al. (2017)
*LAMC2*	1993.41	−2.17	3.90 × 10^−2^	5	LR	Part of Laminino‐332 which is produced by osteoblasts and inhibits osteoclast differentiation	Uehara et al. (2017)
*LGALS1*	9443.16	−1.48	3.40 × 10^−2^	28	LR	Knockout mice have bone loss owing to a lack of bone formation. Promotes bone differentiation of mouse MSCs. Regulates osteoclast activity	Chen et al. (2022), Muller et al. (2019)
*LOXL2*	2208.11.	−7.74	1.10 × 10^−2^	2	LR	Associated with the chondrogenic phase of fracture healing, weak LOXL2 staining found in osteoblasts	Iftikhar et al. (2011)
*LRRN3*	1134.25	1.69	5.00 × 10^−2^	4	HR	Knockout mice have decreased bone mineral density and bone mineral content	Groza et al. (2022)
*MME*	556.80	−2.74	1.10 × 10^−2^	2	LR	A marker of osteoblast differentiation of MSCs	Granéli et al. (2014)
*NR2F1*	776.04	2.99	4.65 × 10^−2^	14	HR	Enhances osteogenic differentiation of human MSCs	Lee et al. (2022)
*NR2F2*	792.09	1.46	2.6 × 10^−2^	1	HR	Knockdown suppresses MSC osteogenic differentiation	Zhu et al. (2016)
*NTRK2*	455.87	−5.19	1.00 × 10^−3^	23	LR	Involved in osteoblast differentiation through ERK signalling	Liu et al. (2018), Zhang et al. (2020)
*OGN*	3654.69	3.32	6.00 × 10^−3^	23	HR	Increases osteoblast differentiation of MSCs through regulation of osteogenic genes; role in senile osteoporosis	Chen et al. (2017)
*PARVB*	119.36	−2.14	1.10 × 10^−2^	28	LR	Knockout mice have increased bone mineral density and bone mineral content	Groza et al. (2022)
*POU3F4*	265.81	9.58	2.00 × 10^−3^	X	HR	Regulates EFNB2 to control temporal bone development	Raft et al. (2014)
*P2RY6*	18.33	−4.67	3.88 × 10^−3^	7	LR	Stimulates bone resorption by osteoclasts. Expressed in osteoblasts	Orriss et al. (2011)
*RBPMS2*	147.30	−1.29	5.00 × 10^−2^	1	LR	Knockout mice have decreased bone mineral density and bone mineral content	Groza et al. (2022)
*ROBO1*	218.37	2.60	2.50 × 10^−3^	26	HR	Slit/Robo signalling regulates bone formation and resorption	Jiang et al. (2022)
*ROBO2*	206.11	3.82	2.00 × 10^−3^	26	HR	Slit/Robo signalling regulates bone formation and resorption	Jiang et al. (2022)
*SENP6*	6868.05	2.36	2.90 × 10^−2^	10	HR	Knockout in osteochondroprogenitors impairs skeletal growth	Li, Lu, et al. (2018)
*SLC2A3*	3592.18	−1.36	2.08 × 10^−2^	6	LR	Silencing in osteoblasts increases proliferation and contributes to glucose uptake	Arponen et al. (2022)
*SLC38A3*	29.53	4.99	1.41 × 10^−2^	16	HR	Heterozygous knockout mice have increased bone mineral density and abnormal bone structure	Groza et al. (2022)
*SMPD3*	315.60	4.48	2.30 × 10^−3^	3	HR	Deficiency in mice results in skeletal deformities and delayed extracellular matrix mineralisation. Role in mineralisation during fracture healing	Manickam et al. (2019)
*SOSTDC1*	80.20	−5.41	2.00 × 10^−3^	4	LR	Involved in bone metabolism, maintenance of bone density and fracture repair	Collette et al. (2016), Tong et al. (2022)
*SOX10*	34.75	5.09	1.77 × 10^−2^	28	HR	Involved in osteogenesis and bone regeneration of mandible	Stuepp et al. (2020)
*TRPM3*	1230.13	7.81	9.39 × 10^−8^	23	HR	Regulates calcium mediated RANKL expression in osteoblasts	Son et al. (2018)

*Note*: BaseMean = average of the normalised count values, divided by size factors and taken over all samples.

Abbreviations: HR, induced pluripotent stem cell (iPSC)‐osteoblasts derived from high‐risk horses; LR, iPSC osteoblasts derived from low‐risk horses; OA, osteoarthritis.

**FIGURE 4:**
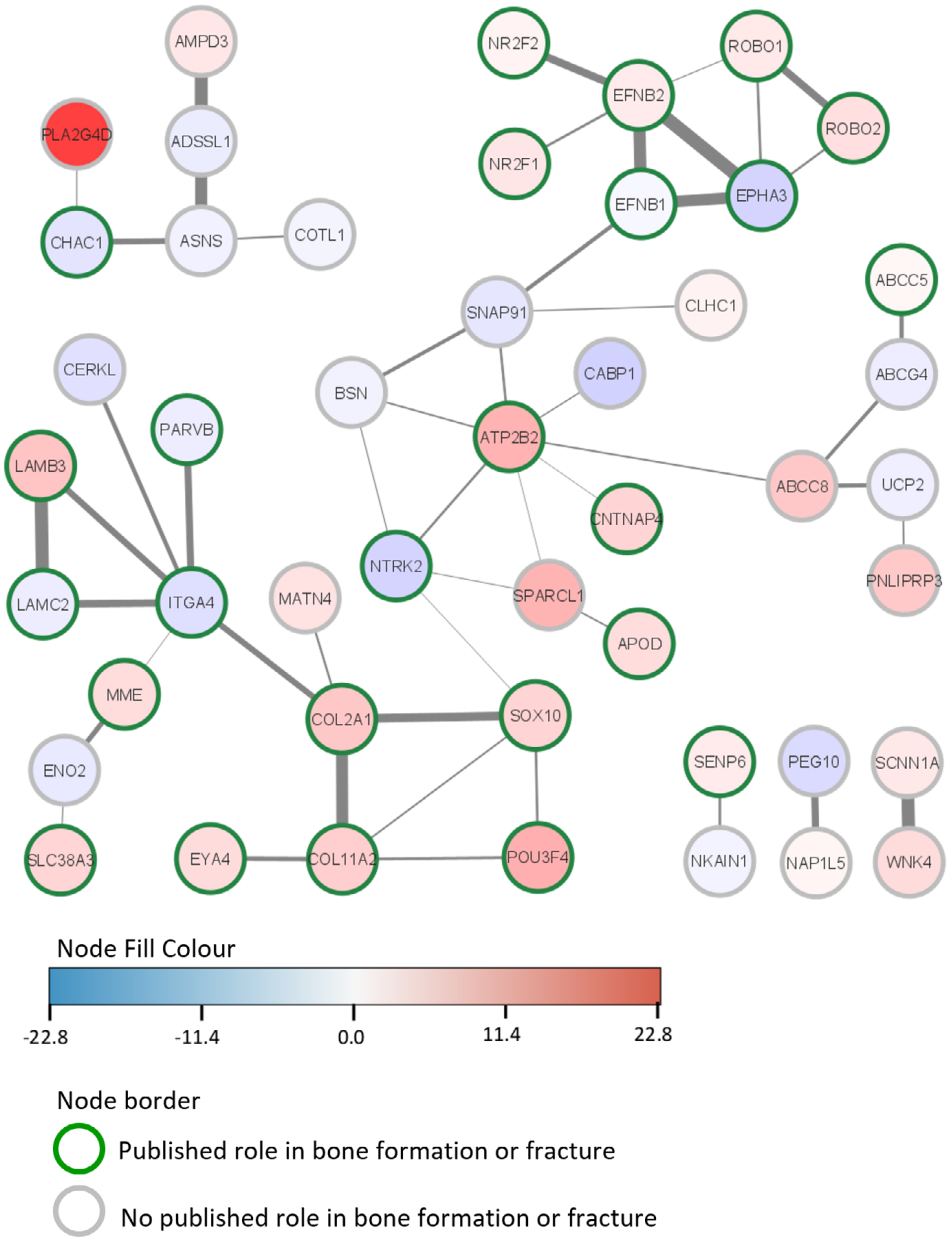
Functional network interactions of the proteins encoded by the 112 differentially expressed genes (DEGs) conducted with string. Proteins encoded by genes which are upregulated in the high‐risk (HR) horses are shown in shades of red; proteins which are downregulated in HR horses are shown in blue. Proteins with a known role in bone formation/fracture have a green border; proteins with no known role in bone formation/fracture have a grey border. A thicker connecting line indicates a stronger interaction score. The DEGs with zero nodes are not plotted.

However, network analysis revealed that there are many interactions between the proteins encoded by genes with known and unknown roles in bone formation and or fracture (Figure 4).

### Functional enrichment of the differentially expressed genes

**FIGURE 5:**
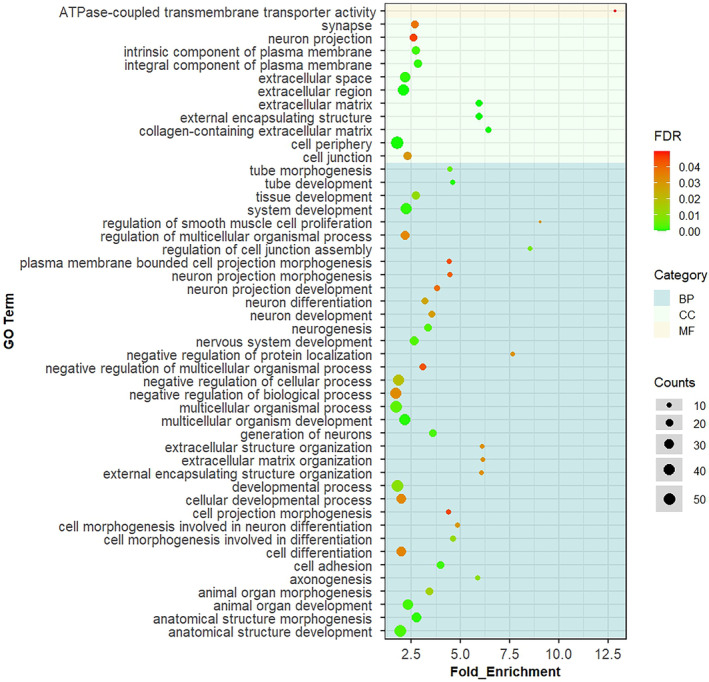
Gene Ontology (GO) terms significantly over‐represented by the differentially expressed genes. The background colour corresponds with biological processes (BP, dark blue), cellular component (CC, light blue) and molecular function (MF, light orange). The colour of the dot shows the significance of the GO terms (colour scale bar indicating the range from 0 [green] to 0.04 [red] false discovery rate). The size of the dot represents the number of differentially expressed genes (DEGs) in that specific GO term.

Gene ontology analysis of the differentially expressed genes demonstrated that they are involved in numerous biological processes including adhesion, development, morphogenesis, differentiation and extracellular matrix (ECM) organisation. Gene Ontology terms associated with development are generally the most significant and have the greatest number of genes associated with them. Those terms associated with the ECM have larger fold enrichments, but fewer genes associated and higher FDRs. However, many of the differentially expressed gene products were also located in the extracellular matrix and for the GO terms associated with cellular compartment the extracellular space, extracellular region and cell periphery were among the most significant and with the highest number of genes associated with them, despite having lower fold enrichments than other terms. The GO term associated with molecular function was ATPase‐coupled transmembrane transport activity, and although this was highly enriched, the number of genes associated with the term was small and the FDR was relatively high (Figure 5).

**FIGURE 6:**
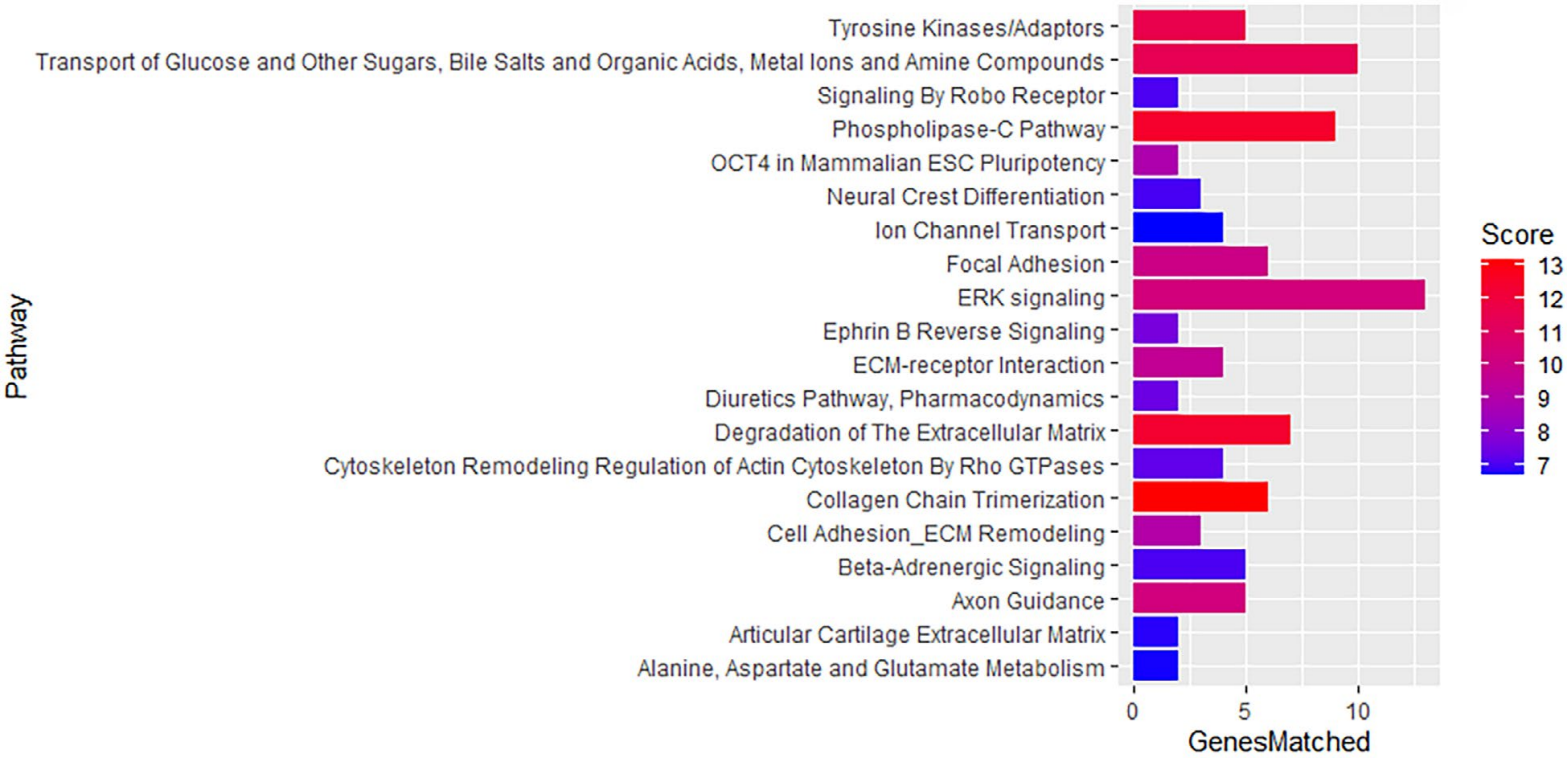
Pathways that are significantly over‐represented by the differentially expressed genes (DEGs). The score is a transformation of the binomial distribution *p*‐value and the score range is divided into three quality levels, according to the *p*‐value they are derived from (after correction for multiple comparisons), represented from lower (blue) to higher (red) score. GenesMatched = number of genes over‐represented in that specific pathway.

Pathway analysis was also performed to identify pathways that are over‐represented by the differentially expressed genes (Figure 6). This revealed a range of affected pathways with extracellular signal‐regulated kinase (ERK) signalling, transport of glucose and other sugars, phospholipase‐C pathway degradation of the extracellular matrix and collagen chain trimerisation having the highest number of genes matched and high scores. Results of the pathway analysis were confirmed using Ingenuity Pathway Analysis (Figure S2).

### Gene set enrichment analysis

As over‐representation analysis was performed on the DEGs only, it excludes genes that did not pass the original cut‐off (*p*‐adj < 0.05, log_2_FC ±1) but that could have a relevant function in a complex disease. gsea associates a disease phenotype (high risk of fracture) to a group of genes/proteins (Mootha et al., 2003; Subramanian et al., 2005). gsea demonstrated that six GO‐based gene sets, six KEGG (Kyoto Encylopedia of Gene and Genomes)‐based gene sets, 11 WP (WikiPathways)‐based gene sets and four REACTOME‐based gene sets, were significantly enriched (Table 3) (FDR < 0.05). Many of the enriched pathways and processes were associated with glycolysis, and the associated genes had a lower expression in the iPSC‐osteoblasts from HR horses.

**TABLE 3:** gene set enrichment analysis (gsea) showing the significantly enriched gene ontology terms and pathways.

Database collections	Gene set	NES	FDR	Higher expression in
*GO‐based list (C5: BP, CC, MP)*				
GOBP_PROTEIN_EXIT_FROM_ENDOPLASMIC_RETICULUM	GO:0032527	2.30	0.037	LR
GOBP_GLYCOLYTIC_PROCESS_THROUGH_FRUCTOSE_6_PHOSPHATE	GO:0061615	2.27	0.033	LR
GOBP_ENDOPLASMIC_RETICULUM_TO_CYTOSOL_TRANSPORT	GO:1903513	2.27	0.026	LR
GOCC_ENDOPLASMIC_RETICULUM_PROTEIN_CONTAINING_COMPLEX	GO:0140534	2.23	0.024	LR
GOBP_NADH_REGENERATION	GO:0006735	2.22	0.027	LR
GOBP_NEGATIVE_REGULATION_OF_NUCLEOTIDE_METABOLIC_PROCESS	GO:0045980	2.20	0.031	LR
*Pathways‐based list (C2)*				
REACTOME_IRE1ALPHA_ACTIVATES_CHAPERONES	R‐HSA‐381070	2.30	0.007	LR
REACTOME_COHESIN_LOADING_ONTO_CHROMATIN	R‐HSA‐2470946	−2.27	0.003	HR
REACTOME_ESTABLISHMENT_OF_SISTER_CHROMATID_COHESION	R‐HSA‐2468052	−2.17	0.012	HR
REACTOME_MITOTIC_TELOPHASE_CYTOKINESIS	R‐HSA‐68884	−2.14	0.012	HR
WP_CORI_CYCLE	WP1946	2.28	0.005	LR
WP_PHOTODYNAMIC_THERAPYINDUCED_HIF1_SURVIVAL_SIGNALING	WP3614	2.27	0.003	LR
WP_GLYCOLYSIS_IN_SENESCENCE	WP5049	2.14	0.011	LR
WP_AEROBIC_GLYCOLYSIS	WP4629	2.13	0.010	LR
WP_GLYCOLYSIS_AND_GLUCONEOGENESIS	WP534	2.05	0.020	LR
WP_BASE_EXCISION_REPAIR	WP4752	2.03	0.024	LR
WP_CLEAR_CELL_RENAL_CELL_CARCINOMA_PATHWAYS	WP4018	1.96	0.041	LR
WP_PHOTODYNAMIC_THERAPYINDUCED_UNFOLDED_PROTEIN_RESPONSE	WP3613	1.96	0.037	LR
WP_ONCOSTATIN_M_SIGNALING_PATHWAY	WP2374	1.95	0.036	LR
WP_MRNA_PROTEIN_AND_METABOLITE_INDUCATION_PATHWAY_BY_CYCLOSPORIN_A	WP3953	1.93	0.044	LR
WP_NIPBL_ROLE_IN_DNA_DAMAGE_CORNELIA_DE_LANGE_SYNDROME	WP5119	−2.03	0.038	HR
KEGG_GLYCOLYSIS_GLUCONEOGENESIS	hsa00010	0.52	0.005	LR
KEGG_INTESTINAL_IMMUNE_NETWORK_FOR_IGA_PRODUCTION	hsa04672	0.55	0.017	LR
KEGG_BASE_EXCISION_REPAIR	hsa03410	0.51	0.012	LR
KEGG_GALACTOSE_METABOLISM	hsa00052	0.54	0.023	LR
KEGG_FRUCTOSE_AND_MANNOSE_METABOLISM	hsa00051	0.49	0.020	LR
KEGG_LEISHMANIA_INFECTION	hsa05140	0.42	0.047	LR

Abbreviations: BP, biological process; CC, cellular component; FDR, false discovery rate; GO, Gene Ontology; KEGG, Kyoto Encyclopedia of Genes and Genomes; MP, molecular process; NES, normalised enrichment score; WP, WikiPathways.

### Weighted gene co‐expression network analysis

**FIGURE 7:**
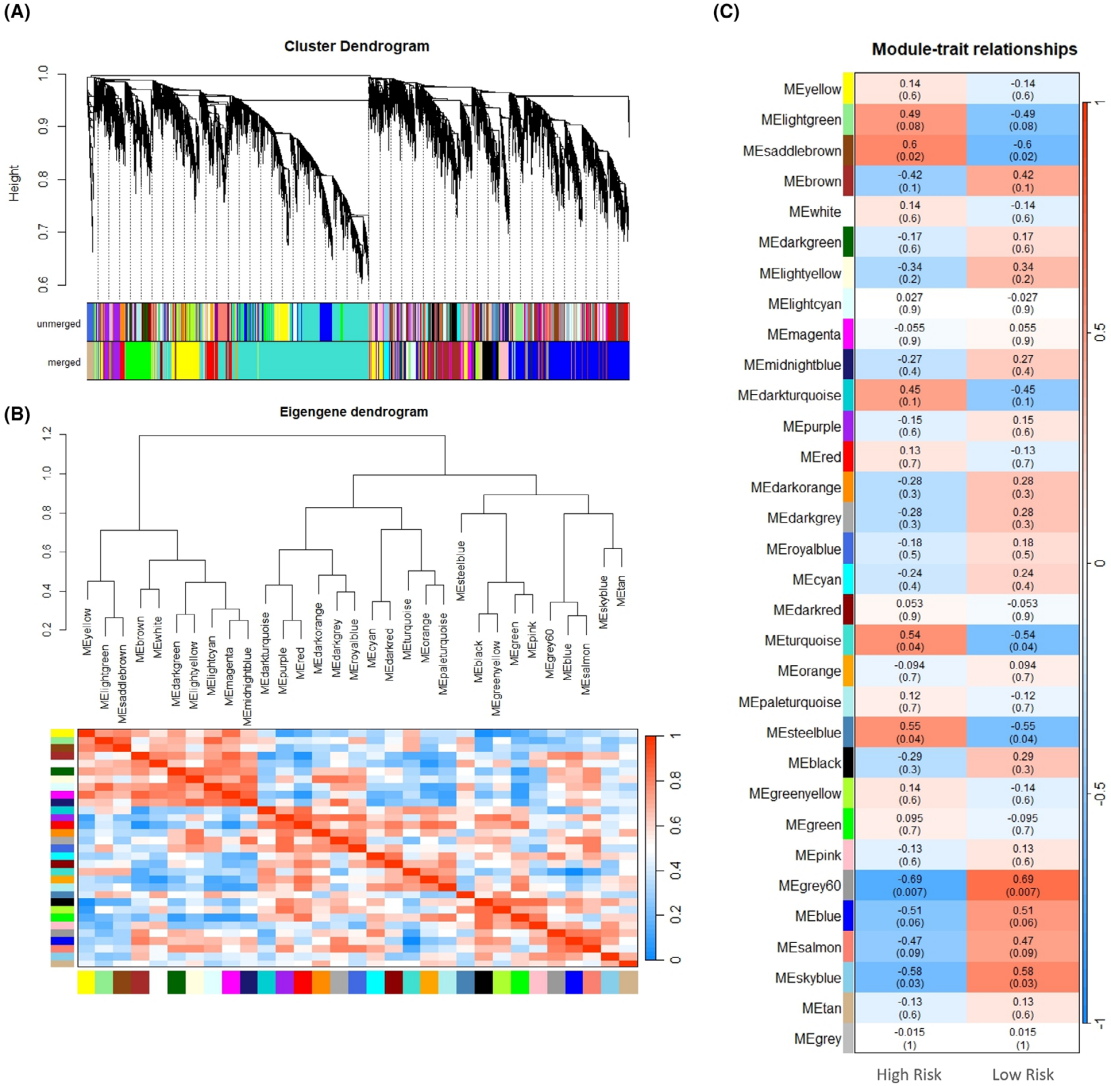
Construction of weighted gene co‐expression network identification of modules. (a) Clustered dendrogram of genes and modules eigengenes; unmerged = preliminary module construction; merged = integrated module. The branches and colour bands represent the assigned module. The tips of the branches represent genes. The distance between two genes is shown as the height on the *y*‐axis. (b) Clustering tree based on modules' eigengenes with the heatmap plot of the adjacencies of modules. Red represents high adjacency (positive correlation) and blue represents low adjacency (negative correlation). (c) Relationships of consensus module eigengenes and risk of fracture. The module name is shown on the left side of each cell. Numbers in the table report the correlations of the corresponding module eigengenes and traits, with the *p*‐values printed below the correlations in parentheses. The table is colour‐coded by correlation according to the colour legend. The intensity and direction of correlations are indicated on the right side of the heatmap (red, positively correlated; blue, negatively correlated).

Weighted gene co‐expression network analysis was performed on all expressed genes, irrespective of whether they were differentially expressed or not. After quality control, this consisted of 13 227 genes across all samples. We identified 32 modules containing highly related genes (Figure 7a,b). Five of these modules were significantly associated with fracture risk (Figure 7c) including the grey60 module (111 genes, correlation co‐efficient (cor) = |0.69|, *p*‐value = 0.007), the saddlebrown module (30 genes, cor = |0.60|, *p*‐value = 0.02), the skyblue module (32 genes, cor = |0.58|, *p*‐value = 0.03), the steelblue module (25 genes cor = |0.55|, *p*‐value = 0.04) and the turquoise module (3879 genes, cor = |0.54|, *p*‐value = 0.04). The 232 genes that could not be included in any module were placed into the grey module and identified as non‐co‐expressed (*p*‐value = 1) (Figure 7c). However, none of these modules demonstrated a strong correlation between module membership and gene significance (0.32–0.49) and hub genes could not be identified (data not shown).

## DISCUSSION

In this study we identified 112 genes that were differentially expressed between iPSC‐osteoblasts derived from horses at high and low genetic risk of fracture. Of these, 27 are unannotated, 43 have published roles in bone formation or fracture and 42 have no known role in bone formation or fracture. However, of these 42 genes, all but five of them have been reported to be expressed in equine bone tissue in publicly available datasets (Kemper et al., 2019; Kuemmerle et al., 2016). Furthermore, STRING analyses demonstrated that 18 of the 42 genes with no known role in bone encode proteins that interact (directly or indirectly) with proteins that have known roles in bone. A further four of the unknown genes encode proteins that interact with each other (SCANN1A with WNK4 and PEG10 with NAP1L5) but have no direct or indirect interactions with proteins that have a known role in bone. This suggests that at least a proportion of these genes are likely to have roles in bone which have not yet been discovered and should be followed up in future work.

### Genes downregulated in osteoblasts from horses with a high polygenic risk score for fracture

Of the top 10 genes with the largest differential expression (DE) that were downregulated in the HR samples, four of them are not yet annotated in the horse genome. However, LOC111769717 is predicted to be *PPWD1*, whose expression is associated with post‐menopausal osteoporosis (Qian et al., 2019). Two of the other non‐annotated transcripts also have predicted genes (*GBA3* and *XDH*), although a role in bone has not been reported. Three of the other top 10 downregulated genes have some previously published links with bone. For example, *SOSTDC1* has been shown to be involved in bone metabolism, the maintenance of bone density and fracture repair (Collette et al., 2016; Tong et al., 2022). *NTRK2* is involved in osteoblast differentiation of mesenchymal stromal cells (MSCs) (Zhang et al., 2020) and binding of NTRK2 to its receptor results in the phosphorylation of ERK1/2, which drives the expression of the bone‐promoting transcription factors *RUNX2* and *SP7* (Osterix) (Liu et al., 2018). *EPHA3* is a member of the ephrin receptor family and ephrin signalling is involved in bone development and homeostasis (Arthur & Gronthos, 2021).

Two of the genes, *INHBE* (a member of the TGF‐*β* superfamily) and *BPIFC* (predicted to enable lipid binding) have no known role in bone formation or fracture, do not appear in the STRING analysis as associating at the protein level with the other differentially expressed encoded proteins, and are not in the gene sets of the enriched GO terms or pathway analysis. Therefore, it is not clear what their biological relevance might be. However, the expression of both genes has been detected in horse bone tissue, and/or cultured horse cells (Kuemmerle et al., 2016). *CABP1* is also in the top 10 and although it has no published role in bone, it appears in the main interaction node of the STRING analysis, is a calcium binding protein and is expressed in horse bone tissue (Kemper et al., 2019; Kuemmerle et al., 2016).

### Genes upregulated in osteoblasts from horses with a high polygenic risk score for fracture

Of the top 10 genes that were upregulated in the HR samples, three were not annotated and, where available, their predicted genes (*MUC2* and *PRR20A*) have no links to bone or fractures. Three of the genes, *PLA2G4D*, *SPARCL* and *ABCC8* have no known role in bone formation or fracture. However, all three genes are expressed in horse bone tissue and/or cultured horse cells (Kuemmerle et al., 2016) suggesting that they may have novel roles in bone which have yet to be defined. ABCC8 functions as a modulator of ATP‐sensitive potassium channels and insulin release and it is in the enriched gene set for the overrepresented pathway transport of glucose and other sugars, bile salts and organic acids, metal ions and amine compounds. Furthermore, it interacts with ATB2BP2, which is also significantly upregulated in the HR samples. SPACRL also interacts with ATB2BP2. *SPARCL*, which is predicted to enable calcium ion, collagen and extracellular matrix binding activity, is a paralogue of *SPARC*, which plays a critical role in bone remodelling (Rosset & Bradshaw, 2016). However, the role of SPARCL in bone is so far unknown. PLA2G4D also appears in the STRING network and interacts with CHAC1. Knock‐out of *CHAC1* in mice leads to decreased osteoblast differentiation and bone density (Crawford et al., 2016). *PLA2G4D* is part of the phospholipase A2 family, and it is present in the enriched gene set for the overrepresented pathway phospholipase‐C pathway.

The remaining five genes of the top 10 that were upregulated in the HR samples include *TRPM3*, which is involved in bone remodelling through the regulation of RANKL expression in osteoblasts (Son et al., 2018). Similarly, *LAMB3* is also involved in bone remodelling. LAMB3 forms part of laminin‐332, which is produced by osteoblasts and supresses osteoclast differentiation (Uehara et al., 2017). *ATP2B2* is involved in maintaining calcium homeostasis. It is upregulated during bone loading (Mantila Roosa et al., 2011), is expressed by osteoblasts (Francis et al., 2002) and is part of a family of proteins which are required for regulating bone mass (Kim et al., 2012). *POU3F4* regulates *EFNB2* to control temporal bone development (Raft et al., 2014). Interestingly, *EFNB2* itself was also significantly upregulated in the HR samples and the ephrin B reverse signalling pathway was significantly over‐represented by the differentially expressed genes.

Therefore, the known functions of the top 10 differentially expressed genes suggest that the regulation of bone remodelling and calcium signalling may be altered in bone cells from horses at high risk of fracture. However, it is possible that the PRS of the iPSCs affects their efficiency of differentiation into osteoblasts and that this accounts for some of the differences in gene expression. Therefore, functional *in vitro* studies are required to confirm the role of the differentially expressed genes in bone and fracture risk and provide biological validation of our results.

### Function of the differentially expressed genes

Gene Ontology analysis of all the differently expressed genes revealed that the molecular function ‘ATPase‐coupled transmembrane transporter activity’ was over‐represented, although despite a high fold enrichment, the number of genes associated with this term was low (four genes: *ABCC5*, *ABCC4*, *ATP2B2* and *ABCC8*) and the FDR is relatively high. However, ATPase activity is crucial for regulating bone formation and resorption (Francis et al., 2002) and *ABCC5* and *ATP2B2* are involved in osteoclast formation and bone homeostasis respectively (Francis et al., 2002; Kang et al., 2020; Kim et al., 2012; Mantila Roosa et al., 2011; Mourskaia et al., 2012). Furthermore, the proteins encoded by all four of these genes interact and therefore further investigation of their role in fracture risk is warranted. Gene Ontology analysis further revealed that the extracellular matrix/region was most often over‐represented by the differently expressed genes. For the biological processes, in addition to the many processes involved in differentiation and development, there were a number of processes related to the ECM, although fewer genes were associated with these terms and they had higher FDR. Nevertheless, for the GO terms associated with cellular compartment, the extracellular space and region had high numbers of associated genes and were among the most significant. Together this may suggest that the differentially expressed genes are ultimately affecting bone homeostasis and ECM components. It is noted that many neuronal GO processes are also over‐represented by the DEGs. The cross‐talk between brain and bone health is becoming increasingly apparent (Otto et al., 2020) and it is possible that many of the DEGs have to date been better studied in neuronal cell types than osteogenic cells, influencing the GO processes they are known to be involved in. For example, Slit/Robo signalling was initially found to be essential during nerve development, before its role in regulating bone formation was discovered (Jiang et al., 2022). Therefore, *ROBO1*/*ROBO2* appear in many of the gene lists associated with neuronal biological processes and pathways.

Pathway analysis revealed multiple pathways involved in bone. For example, the ERK signalling pathway had the greatest number of DEGs and this pathway is involved in fracture repair (Chen & Luan, 2019). Collagen chain trimerisation was one of the most significantly over‐represented pathways and this is affected in numerous bone diseases (Bourhis et al., 2012). Similar to the GO analysis, pathways relating to the degradation and remodelling of the ECM were also over‐represented. The phospholipase C pathway and transport of glucose also had high numbers of genes matched and high scores. The phospholipase C pathway has previously been shown to activate osteoclastogenesis and is involved in fracture repair (Li, Yuan, et al., 2018). As fracture is a complex genetic disease, we also performed gsea and WGCNA on all genes, irrespective of whether they were differentially expressed between HR and LR samples. gsea revealed biological processes and numerous pathways involved in glycolysis. Human patients with diabetes are at higher risk of bone fractures (Valderrábano & Linares, 2018) and altered glucose metabolism may be important in maintaining bone homeostasis (Karner & Long, 2018). Weighted gene co‐expression network analysis identified five modules that were significantly associated with fracture risk with each module consisting of 25–3879 genes. However, none of these modules demonstrated a strong correlation between module membership and gene significance and hub genes could not be identified. We therefore did not perform subsequent gene ontology analysis on the module genes. This probably reflects the small sample size used in the study as it has been suggested that over 20 samples are required for robust WGCNA analysis (Ballouz et al., 2015).

### Limitations of the study

This study had a number of limitations. The polygenic risk score to select cells for use in this study was established previously (Palomino Lago et al., 2024) and it has not yet been validated in a new cohort of fracture cases and controls. Methods of RNA sequencing analysis are undergoing constant improvements, and it is possible that newer approaches to normalisation and differential expression analyses would generate different results (Smid et al., 2018). Furthermore, osteoblasts were only derived from a small number of horses. This may have resulted in a lack of power to detect additional significant DEGs and gene co‐expression networks. However, owing to the samples we had available (Palomino Lago et al., 2024), we were not able to increase the sample size while selecting samples that were at the most extreme ends of the polygenic risk score. Following guidelines on the use of iPSCs for disease modelling, we utilised multiple clones per horse. The number of donors and clones used here is similar to those in other studies of complex diseases in humans (Gorashi et al., 2023; Yde Ohki et al., 2023) and reflects the time and expense involved in deriving and validating an iPSC line. Interestingly a study using iPSCs from three patients with diabetes and three healthy patients (one or two clonal lines from each) found a similar number (245) of differentially expressed genes to us (Gorashi et al., 2023). However, the power to detect statistically significant differences in gene expression is likely to increase if sample size could be increased and recent studies have shown that this will be of more benefit than including more clonal lines from the same individual (Beekhuis‐Hoekstra et al., 2021; Hoffman et al., 2019). Our restricted cohort from which we could select samples also meant that it was not possible to validate the RNA sequencing with qPCR in a larger number of samples. The qPCR performed showed good corroboration of the sequencing results and where differences were observed between the techniques, this was at the level of significance with the same trends in expression patterns being shown for all of the genes examined. Further functional *in vitro* studies are therefore critical to determine gene function and validate our findings. Finally, the original samples were collected anonymously, and the ages and history of the horses were not known. Likewise, for the majority of samples it was not known if the horses were gelded or entire (although two of the HR horses were known to be gelded). During the generation of human iPSCs, the epigenetic clock is reset and is close to zero (Horvath, 2013). This has yet to be confirmed for equine iPSCs; however, it is likely that any variation in the age or work history of the donors does not impact the results.

In our previous study (Palomino Lago et al., 2024), we demonstrated that fibroblast cells differentiated directly into osteoblast‐like cells, with a significant difference in the expression of *COL3A1* and *STAT1* between samples isolated from HR and LR horses (Palomino Lago et al., 2024). In this study, while we saw the same trend for *STAT1*, the difference was not significant (Figure 2 and Appendix S2). The reference transcriptome used did not have *COL3A1* annotated, and therefore it was not analysed in the RNA sequencing. However, qPCR analysis also revealed that while it had the same trend as our previous study (expressed 2.3 fold lower in the HR samples), this difference was not significant owing to the variability between samples. This may reflect the different starting populations of cells and the fact that the osteoblast populations we derived in both studies are likely to be heterogeneous. However, both studies were consistent in demonstrating that *ITGAV*, *CALCRL*, *GULP1*, *SLC401A*, *GLS* and *COL5A2* show no significant differences in expression between the HR and LR osteoblasts. Both studies also demonstrate little or no expression of *MSTN* and *ZNF804A* in the samples. The *COL3A1* and *STAT1* genes lie within a fracture associated region on ECA18 (Blott et al., 2014; Tozaki et al., 2020). In this study, we found only three of the differentially expressed genes to be located on ECA18 (*HOXD10*, *ITGA4*, *CERKL*). However, none of them lie within the associated region. It is not clear if our study lacked the power to detect smaller differences in the expression of any of these genes from this region, or if DNA variants within the region are regulating more distant genes.

Finally, in our model, we directed the iPSCs to differentiate into osteoblasts. However, it is likely that other cell types (e.g. osteoclasts) also contribute to fracture risk. Human iPSCs have successfully been differentiated into osteoclasts (Chen, 2020), but to date this not yet been reported for equine iPSCs and it would be of benefit to measure gene expression profiles in other cell types in the future. Similarly, we did not have access to bone tissue from fracture cases and controls and so were unable to confirm if these differences in expression also occur *in vivo* at either the gene or protein level.

## CONCLUSION

In conclusion, we have demonstrated that iPSC‐osteoblasts derived from horses with high and low polygenic risk scores for catastrophic fracture, have many differently expressed genes that are overrepresented in various pathways and processes that have relevance to bone homeostasis and fracture. A deeper understanding of the consequences of mis‐regulation of these genes and the identification of the DNA variants which underpin their differential expression may reveal more about the molecular mechanisms that are involved in equine bone health and fracture risk. Future work should now perform functional *in vitro* studies to determine the specific roles of these genes in bone and fracture risk.

## AUTHOR CONTRIBUTIONS

**Esther Palomino Lago:** Data curation; formal analysis; investigation; methodology; validation; visualization; writing – original draft; writing – review and editing. **Amy K. C. Ross:** Data curation; formal analysis; writing – review and editing. **Alyce McClellan:** Investigation; writing – review and editing. **Deborah J. Guest:** Conceptualization; funding acquisition; methodology; project administration; resources; supervision; writing – original draft; writing – review and editing.

## CONFLICT OF INTEREST STATEMENT

E. Palomino Lago, A.K.C. Ross and D.J. Guest are affiliated with The Royal Veterinary College, which holds patent WO 2015/019097 ‘Predictive Method for Bone Fracture Risk in Horses’ in relation to this work. This patent claims a method of predicting fracture risk in horses using one or more genetic variations from within the associated region on ECA18. A. McClellan has no competing interests to declare.

## Supporting information


Appendix S1.

Appendix S2.

## Data Availability

The RNA sequencing datasets are available in the National Centre for Biotechnology Information Gene Expression Omnibus repository (www.ncbi.nlm.nih.gov/geo) under accession number GSE255417. The differentially expressed genes and normalised counts data are included in Appendix S2.
